# Evaluation of the sensitivity and specificity of GST-tagged recombinant antigens 2B2t, Ag5t and DIPOL in ELISA for the diagnosis and follow up of patients with cystic echinococcosis

**DOI:** 10.1371/journal.pntd.0008892

**Published:** 2020-11-30

**Authors:** Carlos Sánchez-Ovejero, Eylem Akdur, Raúl Manzano-Román, Ana Hernández-González, María González-Sánchez, David Becerro-Recio, Javier González-Miguel, Okan Akhan, Carmen M. Cretu, Kamenna Vutova, Francesca Tamarozzi, Mara Mariconti, Enrico Brunetti, Ambra Vola, Massimo Fabiani, Adriano Casulli, Mar Siles-Lucas

**Affiliations:** 1 Sierrallana Hospital, Barrio de Ganzo s/n, Torrelavega, Spain; 2 Cukurova Univeristy, Department of Parasitology, Sarıçam/Adana, Turkey; 3 Proteomic Unit, Center for Cancer Research, University of Salamanca, Campus Miguel de Unamuno, Salamanca; 4 Instituto de Salud Carlos III, Centro Nacional de Microbiología, Majadahonda, Madrid, Spain; 5 Instituto de Recursos Naturales y Agrobiología de Salamanca (IRNASA-CSIC), Cordel de Merinas, Salamanca, Spain; 6 Faculty of Medicine, Hacettepe University, Ankara, Turkey; 7 University of Medicine and Pharmacy, Colentina Clinical Hospital—Parasitology, Bucharest, Romania; 8 Specialised Hospital of Infectious and Parasitic Diseases "Prof. Ivan Kirov", Department of Infectious, Parasitic and Tropical Diseases, Medical University, Sofia, Bulgaria; 9 WHO Collaborating Centre for the epidemiology, detection and control of cystic and alveolar echinococcosis, Istituto Superiore di Sanità, Rome, Italy; 10 Department of Clinical Surgical Diagnostic and Paediatric Sciences, University of Pavia, Via Taramelli 5, Pavia, Italy; 11 Department of Clinical Surgical Diagnostic and Paediatric Sciences, University of Pavia, and Division of Infectious and Tropical Diseases, San Matteo Hospital Foundation, Via Taramelli 5, Pavia, Italy; 12 San Matteo Hospital Foundation, Via Taramelli 5, Pavia, Italy; 13 Infectious Diseases Department, Istituto Superiore di Sanità, Rome, Italy; 14 European Reference Laboratory for Parasites (EURLP), Istituto Superiore di Sanità, Rome, Italy; Hitit University, Faculty of Medicine, TURKEY

## Abstract

Cystic echinococcosis (CE) is a neglected zoonotic disease caused by *Echinococcus granulosus* sensu lato. Diagnosis and monitoring of CE rely primarily on imaging while serology is used as a confirmatory test. However, imaging is not always conclusive and currently available serological assays have suboptimal sensitivity and specificity, lack standardization, and are not useful for patients´ follow-up. Seroassays for CE are usually based on hydatid fluid (HF), a complex, variable antigenic mixture, and cross-reactivity exists especially with alveolar echinococcosis. Recombinant proteins based on immunogenic antigens most abundant in HF, such as AgB1, AgB2 and Ag5, have been used to overcome these limitations. None of them so far showed potential to replace HF; however, their performance have been largely tested on a limited number of samples, and comparison of different antigens using the same cohort has been rarely performed. The combination of several immunogenic epitopes in a single recombinant protein could enhance test sensitivity. For the diagnosis and follow-up of patients with CE, we compared the performance of the crude HF, previously described recombinant 2B2t antigen, and GST-tagged version of 2B2t, and novel designed recombinants (GST-Ag5t and the GST-DIPOL chimera containing AgB1, AgBB2 and Ag5 epitopes) by IgG-ELISA format. Samples belong to a retrospective cohort of 253 well-characterized patients with CE, previously described for the evaluation of the 2B2t antigen, 92 patients with alveolar echinococcosis, and 82 healthy donors. The reference standard for CE diagnosis was the presence of a CE lesion as diagnosed by ultrasonography. The highest sensitivity was obtained with HF [86.7%, 95% confidence interval (CI): 81.2–91.0], followed by GST-2B2t (70.0%, 95% CI: 63.1–76.2), 2B2t (65.5%, 95% CI: 58.5–72.0), GST-Ag5t (64.5%, 95% CI: 57.5–71.1) and GST-DIPOL (63.1%, 95% CI: 56.0–69.7). The GST-2B2t had the best specificity (95.8%, 95% CI: 88.3–99.1) and the lowest cross-reactivity (38.7%, 95% CI: 27.6–50.6). Good response to treatment also correlated to negative test results in the GST-2B2t ELISA. While none of the tested recombinant antigen appears suitable to replace HF for the diagnosis of CE, GST-2B2t should be further explored as a confirmation test, based on its high specificity and low cross-reactivity, and for the follow-up after treatment in those patients with positive serology for this antigen.

## Author summary

Cystic echinococcosis (CE) is a neglected parasitic zoonosis. Its diagnosis and follow-up require evaluation with imaging. Currently available serological tests are applied to confirm the diagnosis in doubtful cases, although having limitations in diagnostic accuracy, and they are not useful for patients’ follow-up. Seroassays for CE are usually based on hydatid fluid (HF) obtained from infected animals, with consequent problems of heterogeneity and low specificity. The use of semi-purified HF derivatives or recombinant antigens has been attempted to improve these aspects, but with an unacceptable loss in sensitivity. Most newly developed antigens have been tested on a limited number of samples, not always well characterized, and have been rarely compared using the same samples cohort. Here, we tested and compared three recombinant antigens (2B2t, GST-2B2t and GST-Ag5t), and a recombinant chimeric antigen (DIPOL) based on three highly immunogenic components of HF (B1, B2 and Ag5), in an attempt to increase the sensitivity of recombinant antigen-based seroassays for the diagnosis and follow-up of patients with CE. We found that GST-2B2t had higher sensitivity than the other antigenic preparations, but still not as high as HF, and that GST-2B2t and GST-DIPOL had statistically higher specificity than any of the other tested antigens. GST-2B2t also showed potential for the follow-up of patients with CE after drug treatment.

## Introduction

*Echinococcus granulosus* sensu lato is a cestode with a worldwide distribution mainly transmitted between canids and domestic ungulates. Humans are accidental intermediate hosts (dead-end hosts). Upon ingestion of parasite eggs, larval cysts (metacestodes) grow in different organs, most frequently liver and lung, causing cystic echinococcosis (CE) [1]. CE is a chronic disease, clinically very complex due to numerous variables affecting the course of the disease, including cyst number, size, localization and stage as classified by ultrasonography [2], among others. Ultrasonography is the reference technique for the diagnosis and follow-up of abdominal CE, while serology is usually employed as a confirmatory test. However, this relies mainly on the detection of antibodies against a complex antigenic mixture, the hydatid fluid (HF) obtained from parasitic cysts of animals, which results in suboptimal test performance [3]. Furthermore, no consensus exists on the algorithm to follow when combining imaging and serology, to reach a definitive diagnostic result. A recent work by Vola et al. [4] suggested that the most accurate approach is to apply two first-level tests such as ELISA, immunochromatograpy or indirect haemagglutination, followed by Western Blot in case of discordant results. The recommendation for the use of two (instead of only one) first-level tests, and assessment of their concordant/discordant results, is suggested as a precautionary measure to avoid misdiagnosis, considering the lack of standardization of tests containing HF or its native derivatives.

CE is a neglected disease with limited funding to improve novel diagnostic tools. This has hampered the systematic characterization of antigens that could replace HF in antibody-detection tests [5]. The Human cystic Echinococcosis ReseArch in CentraL and Eastern Societies (HERACLES) project was funded by the European Commission in 2013. One of the main aims of HERACLES was to establish a well-defined collection of parasite and clinical samples, coupled with their respective clinical data [6]. This collection also includes an array of native and recombinant antigens, all from the *E*. *granulosus* sensu stricto G1 genotype (including HF and recombinants malate dehydrogenase [7], actin filament fragmenting protein [8], antigen B1t, antigen B2t [9], and antigen 2B2t [10]). This publicly available resource, named EchinoBiobank, was established at the IRNASA-CSIC in Salamanca, Spain, and is now hosting around 5,000 serum samples suitable for the systematic characterization of diagnostic antigens. A retrospective cohort of serum samples from 253 patients with CE from Italy (Pavia), available in the EchinoBiobank, was previously used for the comparative characterization of HF and the B2t and 2B2t recombinant antigens in ELISA and immunochromatography [10]. We demonstrated that the sensitivity of the tests containing each single recombinant antigen was lower than the sensitivity of the tests based on HF, especially in patients with inactive cysts, while specificity was higher. Furthermore, recombinant antigens showed a limited usefulness for the follow up of patients with CE, mainly due to the high percentage of false negative results against B2t and 2B2t at the beginning of the follow-up [10].

To proceed in the much needed development of a new, better performing test based on recombinant antigens, we produced three recombinant proteins: (i) antigen 2B2t [11] coupled with GST (GST-2B2t); (ii) an N-terminal truncated version of Ag5 (GST-Ag5t); and (iii) a single recombinant antigen combining predicted immunoreactive epitopes from antigens AgB1, AgB2 and Ag5 (GST-DIPOL). The Ag5 is a glycoprotein of 400 kDa, composed of subunits of 57 and 67 kDa that dissociate in subunits of 38 and 24 kDa under reducing conditions, which has been used in its native form to detect antibodies in CE patients with variable success (reviewed in [5]). Ag5 has been shown to be closely related to proteases of the trypsin family regarding structure and sequence, but it lacks protease activity [12]. AgB is a lipoprotein composed of 8-kDa subunits, variably associated oligomers, encoded by a multigene family composed of at least five gene loci (B1–B5) and grouped into five sub-families [13,14]. The role of AgB in parasite biology is still unknown; however, it appears to be involved in several immune modulatory properties (reviewed in [15]). No functional analogues have been described to date for AgB family members or for Ag5, although an “immunological” homologue was found for Ag5, the antigen P29, a somatic antigen of *E*. *granulosus* [16].

The performances of the three new antigens were compared with those of recombinant 2B2t and HF for the detection of IgG in ELISA in samples from patients with CE before and after treatment. For this comparison, we used the same cohort of samples from 253 patients already used in the previous characterization of the B2t and 2B2t antigens [10], together with serum samples from 92 patients with alveolar echinococcosis (AE) and from 82 healthy donors.

## Materials and methods

### Ethics statement

The use and transfer of stocked human serum leftovers from routine analyses carried out in San Matteo Hospital Foundation, Pavia, Italy, was approved by the Ethics Committee of IRCCS San Matteo Hospital Foundation, Pavia, Italy (Acceptance Report 2015041 of 06/07/2015). All samples used in this study were anonymized.

### Study design and samples

A retrospective cohort study was conducted among patients with CE diagnosed and followed-up by ultrasonography (US), based on sonographic pathognomonic signs (US = reference standard; [2]) at the Division of Infectious and Tropical Diseases, San Matteo Hospital Foundation, Pavia, Italy, from 1998 to 2010. Serum samples from CE patients were a convenience sample, including all stocked samples leftover from routine analyses, from patients with CE cysts as diagnosed and staged by US.

**Fig 1:**
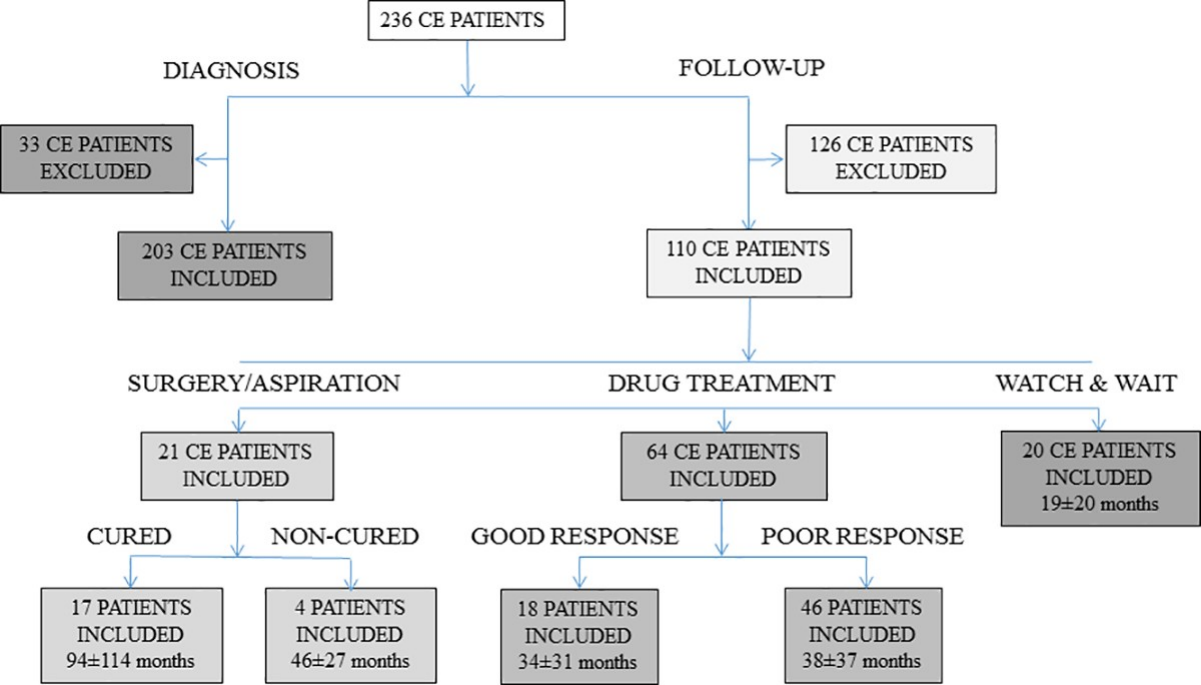
Flow chart showing the participants (cystic echinococcosis patients) in the study. ^1^Patients receiving surgery or percutaneous treatment before the collection of the first serum sample. ^2^Patients with only one serum sample. For the follow-up analysis, patients treated by surgery or aspiration were classified as “cured patients”, if showing no US images suggesting relapses during the follow-up period, and “non-cured patients”, if showing relapses detected by US during the monitoring period. For patients treated with albendazole, patients were classified with good response when the image evolved from active or transitional stages to inactive stages (CE4 and CE5) and with poor response when the image did not change from active or transitional stages to inactive stages. Samples from pre-treatment and from follow-up were collected from the same patients.

Samples from patients with CE used in this study have been described before [10]; demographics and relevant clinical data are summarized in Table 1. A flow chart of participants, including the mean follow-up time with standard deviation (SD) and the number of samples available for each of the study steps is shown in Fig 1. Briefly, to evaluate the sensitivity of the serological tests, the first available serum from patients with CE was used; 203 samples were included (112 collected from patients who were not treated with drugs when the first serum was collected, and 91 collected after drug treatment). The follow-up analysis was carried out based on the classification of patients into clinical groups (surgery-aspiration, drug treatment, and watch and wait approach) and outcomes (“cured vs non-cured” for the surgery-aspiration treatment group and “good vs poor response” after drug treatment), as previously described [10] and detailed below in the Results section. Ninety-two samples from patients with AE donated by Prof. Gottstein (Institute of Parasitology, University of Berne, Switzerland) and 82 sera from healthy donors kindly donated by Dr. Muñoz (University Hospital, Salamanca, Spain) were used to evaluate cross-reactivity and specificity of the different antigens, respectively. Sera from AE patients were a convenience sample from patients diagnosed based on AE-compatible imaging together with a positive Em2^plus^ serological test (Bordier Affinity Products SA, Switzerland) [2,17]. Confirmation of AE and differentiation from CE was based on PCR testing [18] of resected hepatic lesions. AE was chosen for the assessment of cross-reactivity as this is the most relevant parasitic disease in differential diagnosis with CE on imaging [19]. Samples from healthy controls were sera from blood donors collected at the University Hospital in Salamanca (Spain), who fulfilled the requirements for blood donation, including absence of infectious diseases. Although absence of CE was not specifically assessed beyond routine analyses, this samples cohort represents the general healthy population in an endemic area.

**Table 1:** Demographic and clinical characteristics of patients with CE.

Characteristics	Number (percentage)
Gender	
Male	136 (53.7%)
Female	117 (46.3%)
Number of cysts	
0*	33 (13%)
1	142 (56%)
>1	78 (31%)
Median (IQR)	1 (1–2)
Cyst localization	
Liver	231 (91.3%)
Others	18 (7.1%)
NS	4 (1.6%)
Main cyst diameter	
S (0–50 mm)	97 (38.3%)
M (>50–100 mm)	107 (42.7%)
L (>100 mm)	16 (6.3%)
NS	33 (12.7%)
Median (IQR)	59 (45–90)
Cyst classification	
CE1	13 (5.1%)
CE2	20 (7.9%)
CE3a	28 (11.1%)
CE3b	71 (28.1%)
CE4	49 (19.4%)
CE5	39 (15.4%)
NS	33 (13%)

IQR, interquartile range; S, small; M, medium; L, large.

*Patients treated by surgery or aspiration before entering the cohort study. NS, not stated.

### Antigens

Hydatid cyst contents were aseptically collected from *E*. *granulosus* sensu stricto (genotype G1) fertile hydatid cysts obtained from sheep slaughtered as part of the normal work of the Coreses slaughterhouse (Zamora, Spain). The content was centrifuged at 1,000 x *g* for 5 min, and the protein concentration in the supernatant (hydatid fluid: HF) measured with the Micro BCA Protein Assay Kit (Pierce). The HF and the pelleted protoscolices were stored separately at -80°C until used.

The expression vector pGEX-4T1 (GE Healthcare) containing the relevant nucleotide sequences of the antigen 2B2t was used to transform *Escherichia coli* BL21 CodonPlus RIL competent cells (Stratagene). Induction, expression and purification of the GST-2B2t protein were performed as previously described [9,11].

The two new recombinant antigens were produced as follows. The Ag5 sequence with GenBank code JF970202.1 (G1 genotype) was used to design two primers for the amplification of a fragment containing the coding sequence of the antigen except for the first 57 base pairs (Ag5t). These encoded for a signal peptide, as predicted in silico with the PRED-SIGNAL program (http://bioinformatics.biol.uoa.gr/PRED-SIGNAL/input.jsp) that could hamper the subsequent purification of the protein expressed in *E*. *coli*. Primers contained the restriction sequences for *EcoR*I (primer Ag5t-forward 5´- ATT*GAATTC*CTTGGCTTGGAGCTCACTCT) and *Not*I (primer Ag5t-reverse 5´- ATTG*CGGCCGC*TTAGACTGCGTAGCGGTT), indicated in italics in the sequences of each primer. These primers were used for a PCR on cDNA obtained from protoscolices as described before (Hernández-González et al., 2008). PCR was performed for 35 cycles. The ten first cycles consisted of 40 s at 94°C, 40 s at 52°C and 1 min at 72°C, and the next 25 cycles consisted of 40 s at 94°C, 40 s at 60°C and 1 min at 72°C. The PCR product was then processed by electrophoresis in a 1% agarose gel in TBE, followed by excision of the band with the expected molecular weight and purification with the Strataprep DNA Gel Extraction Kit (Stratagene). The purified product was cloned in the pSC-A vector (Agilent) and cloned products used to transform *E*. *coli* cells, following the manufacturer’s instructions. Transformed cells were cultured on agar plates containing ampicillin and single colonies further grown at 37°C in liquid medium with ampicillin. After cells harvesting, recombinant plasmids were extracted using the QIAprep Spin Miniprep Kit (Qiagen), and sequenced at the sequencing service of the University of Salamanca (Spain) using universal primers compatible with the pSC-A vector, to check the cloned sequence and its orientation. The recombinant pSC-A plasmid was then digested with *Eco*RI and *Not*I, and bands were separated in a 1% agarose gel and purified, as described before. In parallel, the vector pGEX-4T1 (Sigma Aldrich) was also digested with *Eco*RI and *Not*I, and the digested vector was processed by electrophoresis and purified following the same procedure. Ligation of the coding sequence of the Ag5t inside the pGEX-4T1 vector, transformation of *E*. *coli* BL21 Condon Plus RIL cells (Agilent) and recombinant protein production was performed as described before [9,11]. The predicted molecular weight of the corresponding recombinant protein was 78.2 kDa (https://www.bioinformatics.org/sms/prot_mw.html). The GST-Ag5t was mainly produced as an insoluble product in inclusion bodies. Therefore, its purification was performed by electroelution. Briefly, cells containing the recombinant protein were harvested at 200 x g for 10 min. Pelleted cells were resuspended in 10 ml phosphate-buffered saline (PBS)/liter of culture and cells sonicated on ice in a VirSonic 300 sonicator (Virtis) at 300 W for 5 cycles of 40 s each. TritonX-100 at a final concentration of 1% was added to the sonicated product and shaken for 30 min at room temperature. The product was then centrifuged at 48,000 x g for 30 min. The resulting pellet was resuspended in PBS containing 1% TritonX-100. Aliquots of 750 μl were boiled for 5 min adding 250 μl SDS sample buffer containing mercaptoethanol and loaded into a single well 12% polyacrylamide gel. Electrophoresis was performed at 30 mA. The gel was then washed thrice for 30 s each with distilled water and stained with CuCl_2_ 0.3M for 5 min with shaking. After further washing as above, the gel was placed on a dark background to observe the bands. The band corresponding to the expected molecular weight was excised and de-stained with EDTA 0.25 M in Tris 0.25M until transparent. The gel strip was then cut into small pieces with a scalpel and the protein electroeluted in a Model 422 Electro-eluter (BioRad), following the manufacturer’s instructions. After elution, an aliquot of the purified protein was checked for purity and integrity by electrophoresis in a 12% acrylamide gel stained with Coomassie Blue. The concentration of the purified protein was calculated in the gel using the Chemidoc XRS scanner (BioRad) by comparison with different concentrations of BSA, and the protein finally stored at -20°C until use.

For the production of the recombinant protein GST-DIPOL, the amino acid (aa) sequences of AgB1 (AF143813.1), AgB2 (U15001.1) and Ag5 (JF970202.1) were subjected to the bioinformatics prediction of their most immunogenic linear epitopes with the SVMTriP program (sysbio.unl.edu/SVMTriP/prediction.php; Yao et al., 2012), based on levels of hydrophobicity, secondary structure prediction and the presence of aa sequences similar to immunogenic epitopes contained in specialized databases. Additionally, alpha-helix regions were found in their aa sequences with the program PreSSAPro (http://bioinformatica.isa.cnr.it/PRESSAPRO/; [20]). These regions act as rigid linkers, facilitating the accessibility of epitopes to antibodies [21]. The selected regions containing the epitopes of interest were amplified from protoscolices cDNA, as described for the Ag5t, by using the following primer pairs: AgB1-forward containing a *Bam*HI site (5´- ACC*GGATCC*TACTTCTTCGAACGTGATCCG) and AgB1-reverse with an *Eco*RI site (5´- ACC*GAATTC*AATCAACCCTCTGAAGTGGGA) for AgB1; AgB2-forward containing an *Eco*RI site (5´- ATT*GAATTC*GATGAGCCAAAAGCACACATGG) and AgB2-reverse with a *Sal*I site (5´- ATT*GTCGAC*ATACTTCTTCAGCACCTCACG) for AgB2; and Ag5-forward (5´- ATT*GCGGCCGC*TGCATCATCAACTCGAAACC) and Ag5-reverse with a stop codon (5´- ATT*GCGGCCGC***TTA**GAAGGTAGGACGGCGG) both containing a *Not*I site for Ag5. Restriction sites are shown in italics and the stop codon in bold. PCR products were purified from agarose gels and subjected to digestion with the corresponding restriction enzymes, as described for Ag5t. The expression vector pGEX-4T3 was digested with *Bam*HI and *Eco*RI, and purified from an agarose gel. This was used for the ligation with the digested PCR product derived from AgB1, and the ligation product was used to transform *E*. *coli* XL1B (Stratagene) cells. Selection of transformants was done as described for Ag5t and sequenced. The vector containing the desired AgB1 fragment was digested with *Eco*RI and *Sal*I and ligated with the digested PCR product corresponding to AgB2 fragment. Selection of clones was done as described above, and the vector containing the expected B1-B2 sequence was digested with *Not*I, dephosphorylated and ligated with the digested PCR product corresponding to Ag5 fragment. The pGEX vector containing the three head-to-tail sequences from specific regions of AgB1, AgB2 and Ag5 was transformed on *E*. *coli* BL21 Condon Plus RIL cells (Agilent) and selected as mentioned, and used for the production of the chimera recombinant protein containing the above-mentioned antigenic regions in one single protein. The predicted molecular weight of the corresponding recombinant protein was 66.6 kDa (https://www.bioinformatics.org/sms/prot_mw.html). The GST-DIPOL protein was insoluble and thus purified by electroelution, as detailed before for the GST-Ag5t. The final product was checked for concentration, purity and integrity as described for the GST-Ag5t protein, and stored at -20°C until use.

#### Test methods

Ninety-six-well polystyrene plates (Corning, Spain) were incubated at 4°C overnight with 2B2t, GST-2B2t, GST-Ag5t or GST-DIPOL (0.5 μg/ml) in carbonate buffer (pH 9.6). Plates were then washed six times with PBS pH 7.4 with 0.05% Tween 20 (washing buffer) and blocked for 1.5 h at 37°C with 200 μl 1% bovine serum albumin (BSA; Sigma Aldrich, Spain) in washing buffer. Sera were then added in duplicate (100 μl/well) at 1:200 dilution in blocking buffer, and plates incubated for 1 h at 37°C. After washing as described above, the secondary antibody (peroxidase-labeled rabbit anti-human IgG, Ref. A8792; Sigma Aldrich, Spain) was added (100 μl/well) at a 1:2,000 dilution in blocking buffer, and plates incubated for 1 h at 37°C. After one further washing as described above, the reaction was developed with 100 μl/well of citrate buffer (pH 5), plus orthophenylene diamine (0.28 mg/ml; Sigma Aldrich, Spain) and hydrogen peroxide (0.4 μl/ml; Sigma Aldrich, Spain). The reaction was stopped with 50 μl/well of 3N sulfuric acid (Panreac, Spain), and absorbance read at 492 nm in an ELISA reader (EAR 400; SLT Lab Instruments, Germany).

The serological index (SI) was calculated for each absorbance (A) value in each individual plate using the following formula: [(NC-S)/(NC-PC)]x100, where NC and PC represent the negative and positive controls, respectively, and S stands for each serum. The negative control consisted of a pool of 10 sera from healthy donors with an A in ELISA of 0.1 to 0.2 and the positive control was a pool of 10 sera from patients with CE with an A in ELISA of 0.4 to 0.5. The SI was used to avoid biases due to assay variability.

#### Statistical analysis of tests diagnostic performance

For all tests, the cut-off value was arbitrarily set at SI = 50. Specificity was calculated as the percentage of negative reactions in serum samples from healthy donors, while cross-reactivity was calculated as the percentage of positive reactions in sera from patients with AE. Sera from patients with AE were specifically used to assess cross-reactivity with the aim of determining the least cross-reactive antigen to support differential diagnosis between CE and AE, which is not always possible or straightforward by imaging alone [19].

Sensitivity, specificity and cross-reactivity of all tests were estimated together with 95% exact binomial confidence intervals (95% CI), and compared between paired samples through the McNemar test. Test sensitivity was assessed using the first time point serum available from each patient with CE, with the exclusion of patients visited for the first time after surgical or percutaneous treatment. Sensitivity was also estimated separately for samples collected before and after albendazole treatment, and according to cysts characteristics (i.e., number, stage, size, and localization). Differences in sensitivity by presence/absence of albendazole treatment and cyst characteristics were evaluated using multivariable logistic regression to account for potential confounders. In patients with more than one cyst, the most active (i.e. likely affecting most the occurrence of a positive serology result) cyst stage classified by US was considered for the analysis [10,22].

#### Statistical analysis of tests performance for patients’ follow-up

The follow-up analysis was conducted considering as baseline: (i) the first serum available after surgical or percutaneous treatment (N = 19 patients, median time between intervention and first serum available 39.5 months, range 6 months to 39 years) or before intervention (N = 2 patients), or (ii) at the first time-point available before the end of the last treatment cycle with albendazole. The first available serum was considered for untreated (watch-and-wait) patients with inactive CE cysts.

The percentage of positive results over time since the start of treatment (≤ 24 months; 25–48 months; > 48 months) was calculated and graphically compared between samples from cured and non-cured patients who underwent surgery/percutaneous treatment, and between samples from patients with good and poor response after drug treatment. Moreover, the percentage over time since first testing was calculated for samples from untreated patients.

Among patients who were positive at baseline, we compared the distribution of the cumulative probability of negativization over time between cured and not cured patients in those who underwent surgical or percutaneous treatment, and between patients with good and poor response in those treated with albendazole. Moreover, we compared the distribution of the cumulative probability of negativization over time among patients who underwent surgical or percutaneous treatment, those with a good response to albendazole treatment, and untreated patients. Differences in the distribution of the cumulative probability of negativization over time among groups were evaluated using the Kaplan-Meier method, and assessed for statistical significance through the log-rank test. Each patient was considered as exposed to negativization from baseline to last test available (for patients who remained positive during follow-up), or from baseline to the first time of negativization (for patients who became negative during follow-up). Time of negativization was estimated by linear interpolation of SI values at the time of last positive test (SI ≥ 50) and time at first negative test (SI < 50). Patients who were tested positive after a previous negative test during follow-up were excluded from this analysis.

Finally, for each test, we used the Wilcoxon rank-sum test to compare the median SI with interquartile range (IQR) between patients treated with albendazole and reaching stable inactivation, at the time they reached inactive cysts stage (CE4 or CE5), and untreated patients with inactive cysts in watch and wait at baseline.

P-values < 0.05 were considered statistically significant. All statistical analyses were performed using Stata 13.1 (StataCorp LP, College Station, Texas, USA).

## Results

### Samples

As described before [10], patients on follow-up with at least two consecutive serum samples (n = 105) were divided in three groups according to their clinical management: (i) surgical or percutaneous treatment, (ii) drug treatment only and (iii) untreated patients with CE4 and CE5 cysts followed by observation with imaging (watch and wait -W&W- approach). Patients in (i) were divided into cured patients if they had inactive/residual lesions at last follow-up visit (n = 17, 55 samples tested with HF-ELISA and n = 16, 53 samples tested with the recombinant antigens in ELISA) and non-cured patients showing recurrence (n = 4, 13 samples). Patients in (ii) were divided into those whose US image changed from active or transitional stages to stably inactive stages (CE4 and CE5) during follow-up in response to treatment (n = 18, 70 samples tested with HF-ELISA and n = 17, 67 samples, tested with the recombinant antigens in ELISA), and those with poor response to treatment, in whom there was no stable change from active or transitional stages to inactive stages (treatment failure) during the follow-up period (n = 46, 186 samples tested with HF-ELISA and n = 43, 172 samples tested with the recombinant antigens in ELISA). Samples from patients with spontaneously inactivated cysts (none of whom had changes in US during the follow-up period), managed by W&W, were also assessed (n = 20, 63 samples for HF-ELISA and n = 20, 59 samples for ELISAs against the recombinant antigens) during the follow-up period.

### Recombinant antigens GST-Ag5t and GST-DIPOL

**Fig 2:**
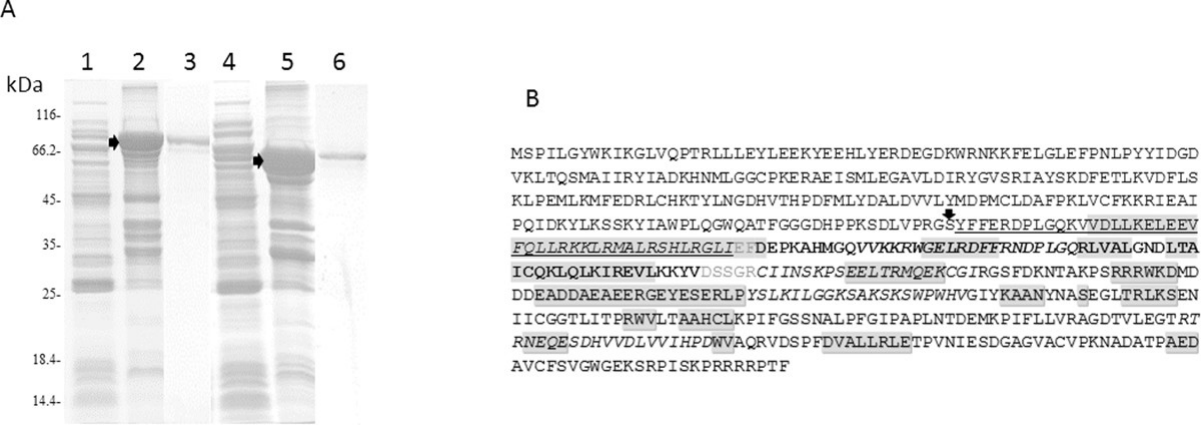
2A. Acrylamide gel stained with Coomassie blue, showing the production and purification results for the recombinant antigens GST-Ag5t (1 to 3) and GST-DIPOL (4 to 6). 1, 4: Culture supernatants; 2, 5: culture pellets, 3, 6: purified proteins. The molecular weights are indicated on the left of the figure, and the position of each recombinant protein is indicated with arrows. 2B. Amino acid (aa) sequence of the recombinant protein GST-DIPOL. The arrow indicates the last aa of the GST tag protein. Sequences corresponding to each antigen are indicated underlined (AgB1), in bold (AgB2) and in shadowed characters (Ag5). Aa in grey correspond to the link between the three antigens. The predicted immunogenic epitopes are shown in italics and the alpha-helix structure is shadowed in grey.

The PCR with the specific primers for the amplification of the Ag5t from protoscolex cDNA resulted in a PCR product with the expected size of 1,418 base pairs (bp). The production of the corresponding recombinant protein fused with GST resulted in an insoluble product of the expected molecular weight (78.2 kDa), as shown in Fig 2. Purification of the GST-Ag5t by electroelution resulted in a yield of 0.6mg/L of culture (Fig 2).

Prediction of the most immunogenic epitopes in the sequences of AgB1, AgB2 and Ag5 resulted in the definition of five different 20 aa-long peptides, one in the AgB1 sequence, one in the AgB2 sequence, and three in the Ag5 sequence (Table 2). Prediction of alpha-helix structure in the corresponding sequences allowed identifying several alpha-helix regions between peptides, between the first peptide and the end of the GST tag sequence, and between the last peptide and the end of the chimera protein. These regions were selected for the specific amplification of the corresponding cDNAs, including two aa stretches at the beginning and at the end of the chimera antigen, to improve the accessibility of the immunogenic peptides to the corresponding antibodies in sera, as shown in Table 2. The final sequence of the chimera antigen is shown in Fig 2. This included the 226 aa of the GST plus 357 inserted aa, yielding a fusion protein of a predicted molecular weight of 66.6 kDa. This was expressed mainly as an insoluble protein, and thus purified by electroelution, resulting in a yield of 0.75 mg/L of culture (Fig 2).

**Table 2:** Amino acid sequences of the antigens B1, B2 and Ag5 of *Echinococcus granulosus* (G1 genotype). The whole amino acid sequence for each antigen is shown. Also for each antigen, the most antigenic epitopes as predicted with the SVMTriP program are underlined; the alpha-helix regions as predicted with the PreSSAPro program are shadowed in grey; and the amino acid sequences corresponding to the cDNAs amplified with the specific PCRs and cloned into the pGEX-4T3 expression vector are in bold. The GenBank accession number for each antigen is indicated between parentheses.

Antigen	Amino acid sequence
Ag B1 (AF143813.1)	MLLALALVSFVVVTQADDGLTSTSRSVMKMFGEVK**YFFERDPLGQKVVDL** **LKELEEVFQLLRKKLRMALRSHLRGLI**AEGE
Ag B2 (U15001.1)	MRTYILLSLALVAFVAVVQAK**DEPKAHMGQVVKKRWGELRDFFRNDPLGQ** RLVALGNDLTAICQKLQLKIREVI**LKKYV**KNLVEEKDDDSK
Ag 5 (JF970202.1)	MARSRPLWIVFVCLFATAALGLELTLDPDELVKAQRESHGGFYFYDSNGA TLMFNRSLFVYRENIYDGWSRWSECSPHTCLEHRYRRCVDDSYTQPVNYL TSSARICPFKYIAEERPCEDKSN**CIINSKPSEELTRMQEKCGIRGSFDKN** **TAKPSRRRWKDMDDDEADDAEAEERGEYESERLPYSLKILGGKSAKSKSW** **PWHVGIYKAANYNA**S**EGL**TRLKS**ENIICGGTLITP**RWV**LT**AAHCL**KPIFG** **SSNALPFGIPAPLNTDEMKPIFLLVRAGDTVLEGTRTRNEQESDHVVDLV** **VIHPDWVAQRVDSPF**DVALLRLE**TPVNIESDGAGVACVPKNADATP**AED**A** **VCFSVGWGEKSRPISKPRRRRPTF**FNPFFWPFGRLWERRPQRPTSLNEIR VSIDPPEKCFHHDDENEAQICAGSSNKGVCAGDTGGGLFCRNEEDGRWYV YGVMGSGPTQYCKSRRWLYNSVGSVIQWINRYAV

### Diagnostic sensitivity, specificity and cross-reactivity, and variables affecting sensitivity

Sensitivity, specificity and cross-reactivity were calculated for each antigen (Table 3). Comparatively, the HF-ELISA showed the highest overall sensitivity (86.7%; 95% CI: 81.2–91.0%), followed by the GST-2B2t-ELISA (70%; 95% CI: 63.1–76.2%), the 2B2t-ELISA (65.5%; 95% CI: 58.5–72%), the GST-Ag5t-ELISA (64.5%; 95% CI: 57.5–71.1%) and the GST-DIPOL-ELISA (63.1%; 95% CI: 56–69.7%). All differences between sensitivity of the HF-ELISA and other tests were statistically significant.

**Table 3:** Sensitivity, specificity and cross-reactivity of the tests for the detection of IgG in ELISA.

		Hydatid fluid	2B2t	GST-2B2t	GST-Ag5t	GST-DIPOL
**SENSITIVITY (overall)**	**n/N** **%** **(95% CI)**	176/203 86.7%^2^^,^^3^^,^^4^^,^^5^ (81.2–91.0)	133/203 65.5%^1^ (58.5–72.0)	142/203 70.0%^1^^,5^ (63.1–76.2)	131/203 64.5%^1^ (57.5–71.1)	128/203 63.1%^1^^,3^ (56.0–69.7)
Before treatment	**n/N** **%** **(95% CI)**	91/112 81.2%^2^^,^^3^^,^^4^^,^^5^ (72.8–88.0)	69/112 61.6%^1^ (51.9–70.6)	70/112 62.5%^1^ (52.9–71.5)	70/112 62.5%^1^ (52.9–71.5)	61/112 54.5%^1^ (44.8–63.9)
After treatment	**n/N** **%** **(95% CI)**	85/91 93.4%^2^^,^^3^^,^^4^^,^^5^ (86.2–97.5)	64/91 70.3%^1^ (59.8–79.5)	72/91 79.1%^1^^,4^ (69.3–86.9)	61/91 67.0%^1^^,^^3^ (56.4–76.5)	67/91 73.6%^1^ (63.3–82.3)
**SPECIFICITY**	**n/N** **%** **(95% CI)**	55/72 76.4%^3^^,^^5^ (64.9–85.6)	56/72 77.8%^3^ (66.4–86.7)	69/72 95.8%^1^^,^^2^^,^^4^ (88.3–99.1)	57/72 79.2%^3^ (68.0–87.8)	64/72 88.9%^1^ (79.3–95.1)
**CROSS-REACTIVITY**	**n/N** **%** **(95% CI)**	64/75 85.3%^2^^,^^3^^,^^4^^,^^5^ (75.3–92.4)	33/75 44.0%^1^ (32.5–55.9)	29/75 38.7%^1^ (27.6–50.6)	37/75 49.3%^1^ (37.6–61.1)	35/75 46.7%^1^ (35.1–58.6)

Statistically significant differences according to McNemar test conducted on paired samples are marked with

^1^ (between the marked tests and the HF-ELISA)

^2^ (between the marked test and 2B2t-ELISA)

^3^ (between the marked test and GST-2B2t-ELISA)

^4^ (between the marked test and the GST-Ag5t-ELISA), and

^5^ (between the marked test and the GST-DIPOL-ELISA).

Specificity and cross-reactivity of each test are also shown in Table 3. The highest specificity corresponds to the GST-2B2t-ELISA (95.8%; CI: 88.3–99.1%), which also showed the lowest cross-reactivity with serum samples from AE patients (38.7%; CI: 27.6–50.6%). Statistically significant differences for the GST-2B2t-ELISA were found in specificity with all tests except for GST-DIPOL-ELISA, and in cross-reactivity with the HF-ELISA.

Clinical variables including cyst stage, size, number and localization, as well as previous treatment with albendazole, were analyzed regarding their association with the sensitivity of each ELISA test. Results are shown in Table 4. The sensitivity of all tests, excluding the GST-Ag5t-ELISA, was influenced by cyst stage, with a higher sensitivity of all tests for patients with active and transitional cysts, compared with inactive cysts. The sensitivity of the HF-ELISA was higher for all cyst stages than the corresponding sensitivity of the ELISAs containing the recombinant antigens (Table 4). Additionally, the HF-ELISA was affected by cyst localization, showing higher sensitivity for hepatic cysts compared with cysts in other localizations, and 2B2t-ELISA was affected by cyst size, with the lowest sensitivity for small cysts (Table 4). Drug administration statistically influenced the sensitivity of both the GST-2B2t- and GST-DIPOL-ELISA, showing a higher sensitivity with serum samples from patients after albendazole treatment (Table 4).

The values of the individual SI for each patient in all tests is shown in S1 Table.

**Table 4:** Sensitivity of the serological tests according to different clinical characteristics.

	Hydatid fluid (ELISA)	2B2t (ELISA)	GST-2B2t (ELISA)	GST-Ag5t (ELISA)	GST-DIPOL (ELISA)
**Serum collection**	P = 0.116^1^	P = 0.280^1^	**P = 0.016**^**1**^	P = 0.811^1^	**P = 0.024**^**1**^
**Before treatment**; n (%)	99 (80.5)	69 (61.6)	70 (62.5)	70 (62.5)	61 (54.5)
**After treatment**; n (%)	89 (91.8)	64 (70.3)	72 (79.1)	61 (67.0)	67 (73.6)
**Number of cysts**	P = 0.484^1^	P = 0.397^1^	P = 0.340^1^	P = 0.787^1^	P = 0.975^1^
**1**; n (%)	117 (82.4)	82 (62.1)	87 (65.9)	83 (62.9)	80 (60.6)
**> 1**; n (%)	71 (91.0)	51 (71.8)	55 (77.5)	48 (67.6)	48 (67.6)
**Cyst stage**	**P = 0.025**^**1**^	**P < 0.001**^**1**^	**P < 0.001**^**1**^	P = 0.515^1^	**P < 0.001**^**1**^
**CE1**; n (%)	13 (100.0)	8 (80.0)	9 (90.0)	8 (80.0)	9 (90.0)
**CE2**; n (%)	20 (100.0)	17 (89.5)	16 (84.2)	14 (73.7)	17 (89.5)
**CE3a**; n (%)	28 (100.0)	23 (85.2)	25 (92.6)	19 (70.4)	22 (81.5)
**CE3b**; n (%)	66 (93.0)	48 (72.7)	54 (81.8)	40 (60.6)	48 (72.7)
**CE4**; n (%)	37 (75.5)	20 (44.4)	21 (46.7)	26 (57.8)	22 (48.9)
**CE5**; n (%)	24 (61.5)	17 (47.2)	17 (47.2)	24 (66.7)	10 (27.8)
**Cyst size**	P = 0.112^1^	**P = 0.043**^**1**^	P = 0.441^1^	P = 0.333^1^	P = 0.091^1^
**Small**; n (%)	66 (78.6)	41 (53.2)	47 (61.0)	46 (59.7)	39 (50.6)
**Medium**; n (%)	93 (88.6)	69 (71.1)	70 (72.2)	61 (62.9)	65 (67.0)
**Big**; n (%)	15 (100.0)	11 (78.6)	11 (78.6)	11 (78.6)	11 (78.6)
**Cyst localization**	**P = 0.017**^**1**^	P = 0.221^1^	P = 0.153^1^	P = 256^1^	P = 0.605^1^
**Liver**; n (%)	177 (87.2)	120 (64.2)	128 (68.4)	118 (63.1)	118 (63.1)
**Other organs**; n (%)	10 (62.5)	12 (80.0)	13 (86.7)	12 (80.0)	9 (60.0)

1 P-values estimated through multivariable logistic regression accounting for the potential confounding due to all variables presented in the table. The multivariable analysis was conducted on 203 and 187 patients with data available for all variables included into the models for HF-Elisa and other ELISAs, respectively. Statistically significant differences are highlighted in bold.

### Follow-up

**Fig 3:**
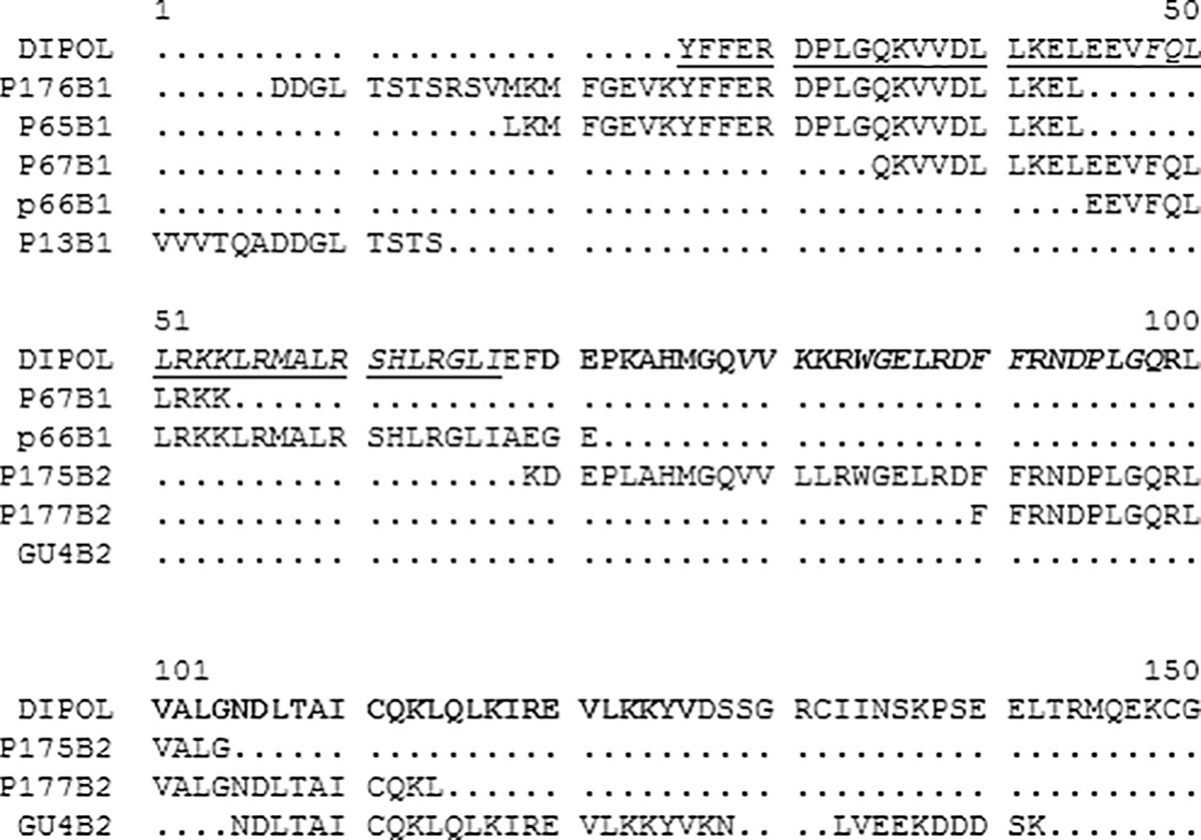
Alignment of the epitopes described by several authors (p65 [35], 1994; p66, p67, p175 and p177 [36]; p13 [37], and GU4 [38]) for the antigens B1 and B2, and the DIPOL sequence corresponding to the abovementioned antigens. The name for each epitope and its antigen of origin are shown in the figure.

The distribution of the cumulative probability of negativization over time of sera from patients who underwent surgery or percutaneous treatment and tested with the different ELISAs was not significantly different among the serological tests under study, regardless of the outcome (Fig 3, panel A). Remarkably, only a small percentage of patients (<50%) classified as cured were positive at baseline against each of the recombinant antigens.

In patients treated with albendazole and classified as good responders to treatment, only tests containing the recombinant antigens GST-2B2t (p = 0.004) and GST-DIPOL (p = 0.017) showed a significantly improved cumulative probability of negativization over time compared with poor responders (Fig 3, panel B). Patients treated with albendazole showed a high rate of positive tests at baseline for all the investigated antigens (>75%).

SI of samples from patients with spontaneous inactive cysts in the W&W group were significantly lower than those of samples from patients with inactive cysts induced by albendazole treatment, except against the GST-Ag5t (Table 5). The distribution of the cumulative probability of negativization over time in patients with good response against albendazole and in patients with inactive cysts in W&W was not significantly different.

**Table 5:** Comparison of the SI of patients with CE4 cysts in non-treated patients and under drug treatment against hydatid fluid, B2t and 2B2t in ELISA.

	PATIENTS UNDER DRUG TREATMENT* (N = 25/23)	NON-TREATED PATIENTS* (N = 24/21)	p-value^1^
Hydatid fluid (ELISA)	283 (252–314)	88 (19–263)	**< 0.001**
2B2t (ELISA)	75 (24–132)	30 (8–57)	**0.013**
GST-2B2t (ELISA)	102 (51–42)	24 (-7–55)	**< 0.001**
GST-Ag5t (ELISA)	79 (47–128)	48 (21–104)	0.133
GST-DIPOL (ELISA)	83 (39–110)	24 (2–59)	**0.007**

* Median and interquartile range (IQR). ^1^ Wilcoxon rank-sum test. Significant P values are marked in bold. N indicates the number of patients tested with hydatid fluid ELISA (first number) or the number of patients tested with the ELISAs containing recombinant antigen (second number).

## Discussion

The new recombinant antigens GST-2B2t, GST-Ag5t and GST-DIPOL were tested here in ELISA for the detection of IgG in sera of patients with CE, together with the previously assessed 2B2t recombinant antigen [9,11], and compared with the results previously obtained with the same cohort against HF [10].

In our study, the GST-2B2t showed a better performance than the 2B2t lacking the GST tag. Higher diagnostic sensitivity of GST-tagged compared to untagged recombinant proteins has been described. Hernández-González et al. [23], using sera from patients with neurocysticercosis and antigen-coated magnetic beads, tested the diagnostic performance of the recombinant antigen T24H tagged with either GST or histidine, finding that the former had better sensitivity. Taken together, these results suggest that the GST tag may allow a more efficient exposure of antigenic epitopes than when the antigens are not tagged or are linked to shorter tails, such as histidine. For what concerns specificity, it has been already shown, in the context of different infectious and parasitic diseases, that GST-tagged recombinant antigens have comparable cross-reactivity, specificity and antigenicity compared with untagged antigens, (e.g. [23–30]). In accordance, the specificity of the GST-tagged 2B2t recombinant antigen was actually higher than the specificity of the non-tagged 2B2t antigen (95.8% vs. 77.8%), and the cross-reactivity with sera from patients with AE was lower for the GST-2B2t compared with the 2B2t antigen (38.7% vs. 44%).

In an attempt to compare the performances of the two major antigens present in the HF, namely AgB and Ag5 [5,31], the new GST-Ag5t recombinant antigen was produced and tested in parallel with the GST-2B2t antigen. Ag5 has been shown to induce antibody responses in patients with CE and constitutes one of the candidates to replace HF in the serodiagnosis of CE. In its native, purified form, Ag5 has shown good performance in the diagnosis of CE [32]. In its His-tagged and maltose-binding protein-tagged recombinant form, Ag5 has been tested for the serodiagnosis of CE by Lorenzo et al [31], showing lower sensitivity than the corresponding purified native antigen (reviewed in [5]). The Ag5 sequence used here corresponds to a protein with a predicted molecular weight of 54.9 kDa, being similar to the His-tagged Ag5 recombinant protein obtained and tested by Lorenzo et al. [31]. Accordingly, the sensitivity and specificity of the recombinant GST-Ag5t (64.5% and 79.2%, respectively) were very similar to those found by Lorenzo et al. [31] with their His-Ag5 recombinant antigen (65% and 89%, respectively). The reactivity of GST-Ag5t was slightly lower than the reactivity of 2B2t and GST-2B2t, and showed lower specificity and higher cross-reactivity than GST-2B2t antigen. The lower reactivity of the recombinant Ag5 tested by Lorenzo et al. [31] compared with the native antigen was attributed to the lack of glycosylated motifs in the protein produced in *E*. *coli*, thought to be important in the triggering of antibody production (reviewed in [5]). This could also be the reason of the only moderate sensitivity of the recombinant GST-Ag5t found here. Differences could also be attributed to the diverse methods used to purify the GST-2B2t and the GST-Agt5, since electroelution can influence the immunogenicity of recombinant proteins, in comparison with affinity chromatography purification (e.g. [33]).

**Fig 4:**
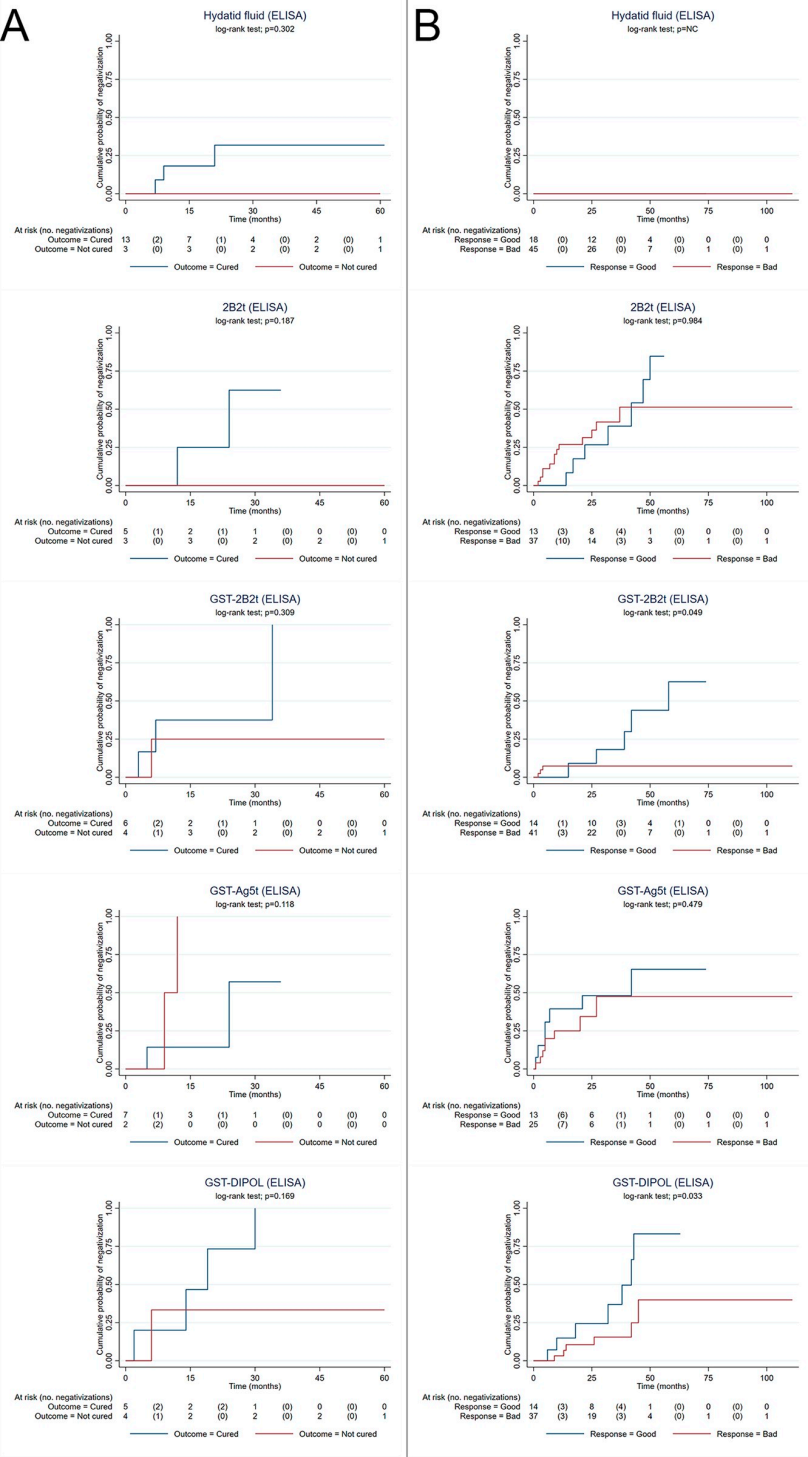
Kaplan-Meier estimates of the cumulative probability of negativization against the different tests over time in: (A) cured (blue line) and not cured (red line) patients who underwent surgical or percutaneous treatment; (B) patients with good (blue line) and bad response (red line) to albendazole treatment. Censored patients/samples, these can be derived by difference, and were due to missing attendance to the follow-up visit or no availability of serum leftover from routine serology to be stored for biobanking.

Differential expression of antigens by different cyst stages has been approached using proteomics by Ahn et al. [34]. They found a change in the immunoproteome profile of the HF, including differential recognition of Ag5 and AgB proteoforms, when sera from patients with CE cysts in different stages were tasted with HF obtained from CE cysts in different stages. Peptides with diagnostic potential described by other authors and derived from AgB1 or AgB2 are shown in Fig 3. These include peptides p65 [35], p176, p66, p67 [36] and p13 [37] from *E*. *granulosus* AgB1, and peptides p175, p177 [36], and GU4 [38] from *E*. *granulosus* AgB2. These publications describe peptides designed without using any algorithm of immunogenicity prediction, thus their usefulness for CE diagnosis had to be empirically tested. Only p65 and p176 from AgB1, and GU4, p175 and p177 from AgB2 were tested as synthetic peptides in ELISA for the detection of total IgG in sera from patients with CE by several authors, with sensitivity and specificity ranging from 12% to 80% and from 80% to 100%, respectively (reviewed in [5]). Taken together, these studies support the hypothesis that the detection of a single parasite antigen and the use of single peptides may not be enough to diagnose CE in every case, showing the need for using several peptides in combination to try improving test sensitivity. The production of a chimera protein containing several epitopes would save production costs [39–41]. Therefore, in an attempt to improve the sensitivity of the single recombinant antigens, we selected the most immunogenic linear epitopes of the three main HF antigens (AgB1, AgB2 and Ag5), and combined them in a single recombinant protein. For comparative purposes, the above-mentioned peptides are shown aligned with the AgB1 and AgB2 regions included in our GST-DIPOL recombinant protein (Fig 4). The *in silico* prediction of immunogenic (linear) epitopes does not guarantee the best immunogenicity in nature. This indeed seems the case for the epitopes included in DIPOL. The DIPOL chimera antigen showed lower sensitivity than HF and other single recombinant antigens. While cloning of the epitopes flanked with rigid linkers (alpha-helix structure) should increase the chance of the epitopes to be available to react with their specific antibodies (reviewed in [42]), interferences in epitope recognition due to conformational hindrances cannot be ruled out. DIPOL reactivity could have also been hampered by its insolubility, needing purification by electroelution as opposed to affinity chromatography.

Our results show that cloning of tandem repeats of a single antigen (2B2t) results in better sensitivity in an ELISA for the diagnosis of CE, than a chimera recombinant antigen including *in silico* predicted immunogenic fragments of three different antigens (AgB1, AgB2, and Ag5). While no tested recombinant antigen appears suitable to replace HF for the diagnosis of CE, GST-2B2t should be further explored as a confirmation test, thanks to its high specificity and low cross-reactivity, especially in CE-AE co-endemic areas. Furthermore, we have also confirmed that cyst stage and previous drug administration influence the sensitivity of serological tests [10,11,22]. On the contrary, variables such as cyst number or cyst size, found important for the tests result in previous studies [11,22], seem not to be consistent to our findings.

Besides the diagnosis of infection, an important point of concern in the clinical management of patients with CE is their monitoring over time. We have shown here and previously [43] that a drop in antibody titers against specific recombinant antigens could indicate success after surgery or percutaneous treatment. However, the evaluation of their usefulness was seriously hampered by the low number of patients with a positive test at admission, likely due to the extremely variable, often long time between intervention and first serum available for the study. Usefulness for follow-up was better investigated in patients treated with drugs, who showed and acceptable rate of seropositivity at baseline, and where decline in antibodies against GST-2B2t and DIPOL showed a good correlation with response to treatment. The practical application of serial tests against these antigens for the follow-up of drug-treated patients should be further investigated through studies based on a broader cohort, possibly including patients with regular follow-up intervals.

This study has several limitations. Firstly, the sample size did not allow detecting statistically significant small differences in sensitivity/specificity of the investigated serological tests. In fact, assuming a reference 5% increase in sensitivity and specificity, we found that the statistical power to detect such minimum increase as statistically significant was in the range 27–36% for sensitivity and 15–17% for specificity. However, deciding on a clinically relevant difference in a serological test is not straightforward. In CE, where the “weight” of a false positive vs. a false negative result depends on the stage of the cyst and the type of differential diagnoses for each cyst stage. An additional limitation of our study is the heterogeneity of the samples population, due to the complexity and heterogeneity of CE clinical presentations. This did not allow performing the analysis of predictive values, which should be calculated for each specific clinical setting. Additionally, a potential bias could have been derived from the compliance of people with a less favourable outcome to present to follow-up visits, compared to people with good prognosis. This would results in more follow-up samples, like we have seen in the drug-treated group in our cohort. This bias would have reduced the power to detect a significant difference between groups at follow-up. Finally, cross-reactivity with other parasitoses in our study was assessed only for AE. At present, the WHO-IWGE expert group recommends that serology must be applied only when a lesion suspect of CE is visualized on imaging. Such differential diagnosis, in the case of parasitic infections, applies mainly for AE and, in particular circumstances, for cysticercosis. In any case, cross-reactivity with other helminth infections commonly present in CE endemic areas is important, but unfortunately such sera were not available. The biobank collection built up during the HERACLES project [6] is one of the few examples of biobanking of human samples and associated clinical data in the field of parasitology. Further validation of the recombinant antigens used here and of other new diagnostic tools to be developed in the future for the diagnosis and follow-up of CE should be ideally extended with using samples collected in biobanks devoted to other helminth infections commonly present in CE endemic areas. Thus, contribution of other researchers and clinicians for the collection of those samples and associated data is encouraged.

In summary, the use of the GST tag enhanced the sensitivity and specificity of the 2B2t antigen, while the combination of several epitopes of AgB1, AgB2, and Ag5 in a single recombinant antigen did not show better performance than the AgB2 antigen cloned in tandem. While no tested recombinant antigen appears suitable to replace HF for the diagnosis of CE, GST-2B2t should be further explored as a confirmation test, thanks to its high specificity and low cross-reactivity. Additionally, the GST-2B2t antigen is promising and deserves further assessment for the follow-up of patients with CE after treatment.

## Supporting information

S1 STROBE Checklist (DOC)

S1 Table (XLSX)
